# Diagnosis and Treatment of Obstetric Anal Sphincter Injuries: New Evidence and Perspectives

**DOI:** 10.3390/jcm10153261

**Published:** 2021-07-23

**Authors:** Antonino Spinelli, Virginia Laurenti, Francesco Maria Carrano, Enrique Gonzalez-Díaz, Katarzyna Borycka-Kiciak

**Affiliations:** 1 Department of Biomedical Sciences, Humanitas University, Via Rita Levi Montalcini 4, 20090 Milan, Italy; virginia.laurenti@humanitas.it (V.L.); francesco.carrano@humanitas.it (F.M.C.); 2 IRCCS Humanitas Research Hospital, Via Manzoni 56, 20089 Milan, Italy; 3 Pelvic Floor Unit, Department of Obstetrics and Gynaecology, Complejo Asistencial Universitario de León (CAULE), C/Altos de Nava S/N, 24080 León, Spain; enriquegonzalezdiaz@hotmail.com; 4 Department of Obstetrics and Gynaecology, Complejo Asistencial Universitario de León (CAULE), C/Altos de Nava S/N, 24080 León, Spain; 5 Department of Colorectal, General and Oncological Surgery, Centre of Postgraduate Medical Education, 80, Ceglowska Street, 01810 Warsaw, Poland; katarzyna.borycka@oasis-diagnostics.eu

**Keywords:** obstetric anal sphincter injury (OASI), anal sphincter, perineal tear, faecal incontinence, diagnostics, treatment

## Abstract

Perineal injury during childbirth is a common event with important morbidity associated in particular with third-and-fourth degree perineal tears (also referred to as obstetric anal sphincter injuries—OASIS). Early diagnosis of these damages is mandatory to define a prompt therapeutic strategy and thus avoid the development of late-onset consequences, such as faecal incontinence. For this purpose, various diagnostic exams can be performed after a thorough clinical examination. The management of OASIS includes several measures and should be individualized according to the timing and features of the clinical presentation.

## Key Points

Obstetric anal sphincter injury affects about 5.7% of primiparous women who deliver vaginally.

Most women who sustain OASIS remain asymptomatic, but some of them are at risk for developing incontinence as they age or with future deliveries.

Affected women should be referred to a perineal clinic for further investigation, including both traditional and innovative diagnostic techniques.

When OASI is diagnosed immediately after vaginal delivery, primary repair represents the mainstay of treatment.

Faecal incontinence resulting from an undiagnosed OASI can be addressed by both conservative and operative measures.

## 1. Introduction

Traumatic injuries to the anus and perineum can occur for several reasons, including pelvic trauma, sexual intercourse, and iatrogenic injuries, and potentially lead to sphincteric damage.

In women, vaginal delivery represents the most common cause of perineal trauma [1].

While anterior perineal trauma, involving the labia, anterior vagina, urethra, or clitoris, is usually associated with little morbidity, posterior perineal trauma, which accounts for any injury to the posterior vaginal wall, perineal muscles or anal sphincter, can lead to more complex damages [1,2].

Perineal damages following labour and vaginal delivery are mainly sustained by two different mechanisms of injury: tears of the perineal striated muscles and neurogenic damage of the pelvic floor nerve supply.

The stretching and compression of the birth canal occurring during the descent of the foetus may lead to demyelination and subsequent denervation of pudendal nerves [3].

The diagnosis of neurogenic injuries is challenging. In the assessment of a sphincter trauma without any damage occurring to the muscles, imaging methods, such as endoanal ultrasound (EAUS) or magnetic resonance imaging (MRI), are of poor utility. Pudendal neuropathy can be detected by neurophysiologic tests, including concentric needle electromyography (EMG) and pudendal nerve motor latency, but their routine use is not recommended, as it does not impact treatment [4]. Indeed, most neuromuscular injuries resolve spontaneously during the first year after delivery [5].

Post-delivery muscular damages are known as obstetric anal sphincter injuries (OASIS).

OASIS are also referred to as third- and fourth-degree spontaneous perineal lacerations, according to the currently accepted classification [6].

Despite the increasing attention on OASIS prevention, a noticeable number of vaginal deliveries is still complicated by these damages. The reported incidence of OASIS ranges between 0.5% and 17% [7,8,9,10,11,12,13,14,15], though it is not reliably established due to differences in coding and reporting. A much higher risk is observed among primiparous women (5.7%) than in multiparous ones with no prior OASIS (1.5%) [16].

Spontaneous vaginal delivery increases the risk of pelvic floor dysfunction, which is the combination of some or all of the following conditions: urinary and faecal incontinence and pelvic organ prolapse. Conversely, Caesarean section seems to play a protective role in the long term [17].

Caesarean delivery has been shown to provide partial protection for pelvic organ prolapse and, to a lesser degree, urinary incontinence. Nevertheless, delivery exclusively by Caesarean has not as yet been shown to be protective for FI in comparison with vaginal delivery [18,19,20].

Furthermore, Caesarean section has been associated with increased risks for fertility, future pregnancy, and long-term childhood outcomes [20].

For these reasons, when involving women in the decision making on their mode of delivery, it is of uttermost importance to identify those mothers least susceptible to birth injury and those at high risk of incurring physical damage to their sphincters [19,20].

**Figure 1:**
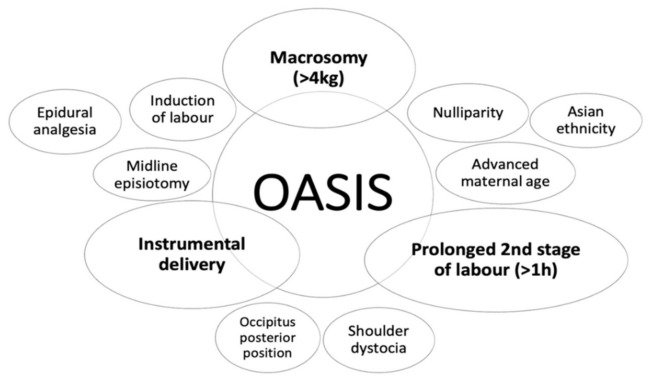
Acknowledged risk factors for the development of OASIS.

Established risk factors for OASIS development (Figure 1) include high infant birth weight (>4 kg), prolonged second stage of labour (>1 h), and instrumental vaginal delivery (particularly by forceps). Nulliparity, advanced maternal age, Asian ethnicity, persistent occiput posterior position, induction of labour, epidural analgesia, shoulder dystocia, and midline episiotomy (associated with a higher incidence of sphincter injury than a mediolateral incision) [21] have also been described as independent risk factors for the development of obstetric anal sphincter injury even if not consistently [22].

Unfortunately, most of these risk factors are not modifiable. Some potentially protective measures have been proposed, including intrapartum obstetric manoeuvres. Manual perineal protection and warm compression on the perineum continuously in the second stage of labour appear to reduce the risk of OASIS [23].

The role of episiotomy in preventing OASIS and/or anal incontinence is still a matter of debate, but at present, routine episiotomy is not recommended [24].

**Figure 2:**
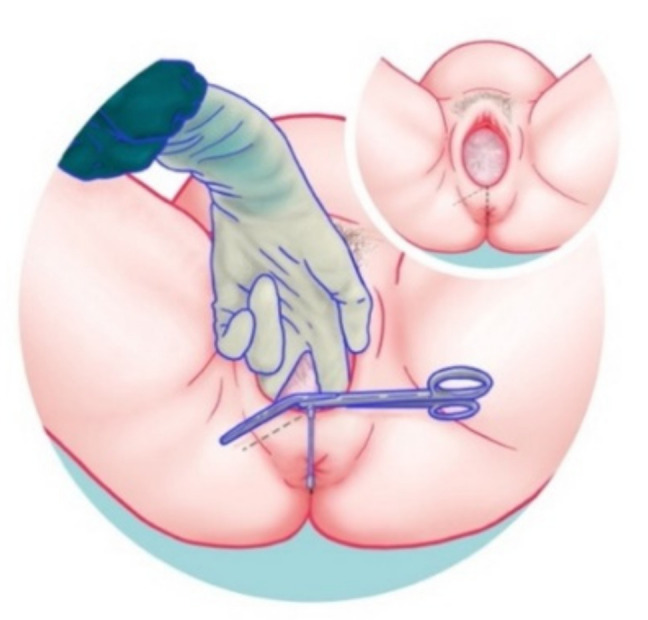
Mediolateral episiotomy with an angle of 45–60 degrees from the midline.

In instrumental deliveries or whenever indicated, a mediolateral episiotomy with an angle of 45–60 degrees from the midline should be considered (Figure 2) since it is associated with a lower incidence of sphincter injuries than spontaneous lacerations [1,17].

OASIS represents the leading cause of anal incontinence (AI) in women and severely burdens the individual on a psychological, physical, and social level [25].

Moreover, OASIS can lead to short-term consequences, including wound hematoma and wound breakdown, abscess, and recto-vaginal fistulae formation, and to later symptoms, like perineal pain and dyspareunia. Persistent pain and discomfort from perineal trauma may also cause urinary retention and defecation problems.

Although most women experiencing sphincter damage during childbirth may remain clinically silent, some of them are at risk for developing incontinence as they age or with future deliveries. To prevent the long-term complications of an undiscovered sphincter injury, effective tools allowing early diagnosis and, therefore, more effective treatment are needed.

We aim to provide a review of the diagnosis, management, and treatment of OASIS, focusing on the most recent detective tools and strategies of cure.

## 2. Diagnosis

Since all women having a vaginal delivery are at some risk of sustaining perineal tears, a systematic physical examination should be performed in the immediate post-delivery setting.

Accurate clinical assessment, including an inspection of the perineum and a digital rectal exploration, has thus far remained the most appropriate diagnostic tool for early detection of OASIS [4]. However, up to 80% of sphincter injuries seems to be missed in the delivery suite [26,27].

Appropriate sensibilization and training of childbirth professionals remains the mainstay for correct detection of OASIS following delivery. A proper detection of these lesions may depend on the experience of the assessor; structured hands-on workshops are important to improve health professionals’ abilities to identify and repair the internal sphincter correctly [28,29,30].

Acceptance that injury is not a medical mistake but often an inevitable complication of childbirth is mandatory in order to let professionals do their best to detect and fix it.

The frequency of undiagnosed injuries also underscores the need for imaging in the evaluation of the anal sphincter.

### 2.1. Diagnostic Tests

More advanced diagnostics (Table 1) are not usually performed during the immediate post-delivery period and are often delayed until some weeks after the injury. This is mainly because of the extensive knowledge and experience needed to detect anal sphincter injuries with these tools and the limited availability of equipment in the setting of obstetric wards [31].

The role of imaging in the early diagnosis of OASIS is further limited by the risk of incorrect interpretation of images and consequent potential overestimation of their occurrence [28,29,32]. Indeed, the detection rate of OASIS has significantly increased since the introduction of EAUS in the assessment of the anal sphincter following vaginal delivery (ranging from 11% to 36% of women), but it is unclear whether these findings represent clinically relevant defects [33].

There is no consensus as to which women benefit from additional imaging; imaging decisions are based on provider preference and equipment availability.

#### 2.1.1. Endoanal Ultrasound (EAUS)

EAUS represents the gold standard method for the detection of both external and internal anal sphincter injuries as well as for the evaluation of the injury site and extent of damage [34].

Three-dimensional EAUS (3D-EAUS) permits the detection of even small sphincter injuries otherwise invisible or misinterpreted [35,36].

EAUS is an accurate, highly sensitive, and safe way of assessing the perineum for OASIS, with none or minimal discomfort for the mother (depending on the size of the probe chosen for the exam) [37,38,39].

It also allows to assess which injuries are more likely to benefit from a surgical repair and to identify the exact location of the snapped ends of the anal sphincter, potentially playing a role in the intraoperative setting.

Though its role has not been clearly determined yet, EAUS can be used in the early follow-up after anal sphincter repair for OASIS to identify patients at higher risk of long-term complications who may benefit from early referral to specialist centres [38,40].

It may also serve as a helpful tool to plan the mode of delivery in women at risk of developing incontinence with future pregnancies [41,42].

#### 2.1.2. Transperineal Ultrasound (TPUS)

**Figure 3:**
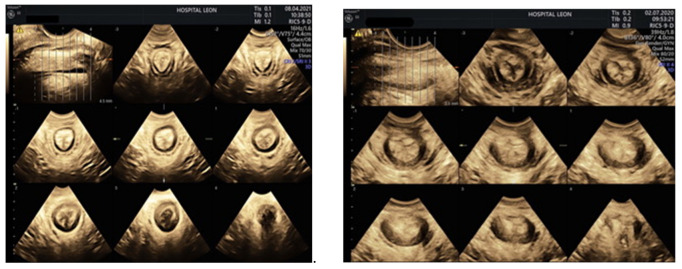
Tomographic ultrasound imaging of internal (IAS) and external anal sphincter (EAS). Longitudinal plane encompassing entire length of EAS is used as reference frame (upper left image of both pictures). The EAS appears as a ring of hyperechogenicity surrounding the thin hypoechogenic ring of the IAS. The mucosal folds of the anal canal appear in the center of the image as the innermost layer. Sphincters are normal in all the transverse slices of the left picture (complete rings). The picture on the right show a full-thickness defect of both sphincters (both concentric rings are interrupted).

TPUS consists in the application of a high-frequency curved array transducer on the perineum (transperineally or translabially) to assess the functional anatomy of the pelvic floor and detect anal sphincter abnormalities. Figure 3 compares TPUS images of a normal anal sphincter complex and those of a damaged one.

In comparison to EAUS, it has the advantages of being more accessible, less invasive, and more acceptable to the patient, being exo-anal. For such reasons, TPUS has become increasingly popular in recent years [41,43,44]. On the other hand, the lack of panoramic view and worse tissue discrimination when compared to EAUS have so far prevented a wider spread of this method in clinical practice [27,45,46,47].

Even though TPUS has not yet achieved the same results in terms of images quality when compared to the current gold standard technique, more and more studies are observing a good correlation between these two techniques [48].

Regardless of the adopted approach, the use of ultrasound diagnostics in the obstetric wards is significantly limited by the inter-observer variability and the extensive knowledge and experience needed to detect anal sphincter injuries with these methods.

#### 2.1.3. Magnetic Resonance (MRI)

The use of both endoanal and external MRI to detect anal sphincter injuries has been investigated. When compared to EAUS, endoanal MRI demonstrates a lower sensitivity in diagnosing internal anal sphincter defects, while the two methods are comparable for the assessment of the external anal sphincter [49,50,51].

A recent study comparing external phased array MRI with EAUS observed that the former is more sensitive in detecting external anal sphincter defects and represents a feasible tool in the diagnosis of OASIS [52].

MRI has the advantages of being non-invasive, reproducible, not operator-dependent, and providing objective findings, thus allowing comparison between study populations.

In centres where EAUS is not available while MRI is, it may help in making the diagnosis, particularly if there is concern for fistula [52].

In the study of pelvic floor disorders in general, dynamic MR defecography represents another reliable option for non-invasive evaluation of the entire pelvic floor.

For anal incontinence alone, most of the times caused by direct sphincter laceration or indirect nerve damage from previous vaginal delivery, MR defecography has little to offer besides showing inability to retain the rectal contrast [53].

Therefore, for anatomic OASIS detection, pelvic MR or ultrasonography are better suited [54], while for functional evaluation, anorectal manometry is the gold standard, as reported below.

However, the use of MR defecography could be justified during the study of patients presenting with faecal incontinence long after delivery to investigate whether it could be ascribed to an end-stage descending perineal syndrome, which would change the surgical approach [55].

#### 2.1.4. Anorectal Manometry

Anorectal manometry represents the gold standard for assessing anal sphincter function [56]. It is performed with either an air- or water-charged catheter system; it measures anal length and the pressure in the anal canal at rest and during voluntary squeeze.

Anorectal physiology testing detects absent or impaired anal sphincter function, which can indicate prior sphincter trauma, but such findings are not exclusive to OASIS.

Until recently, the lack of standards for both examination performance and the interpretation of the results constituted an important issue related to this exam. However, the International Anorectal Physiology Working Group (IAPWG) has just solved this problem, publishing a standardized protocol for the preparation and performance of anorectal function testing and a language to describe the results of diagnostic tests (London Protocol), which is currently in force [57].

Now, the examination’s inconvenience remains its length, nuisance, and low availability, as it is a highly specialized diagnostic.

#### 2.1.5. Impedance Spectroscopy

The need for a simple, useful tool for early diagnosis, usable even by untrained operators, remains unmet. Recently, an option proposed to address this problem is impedance spectroscopy.

The electrical impedance is physical property of tissue and, in the perianal area, can reflect the condition of the anal sphincter from both anatomical and functional perspectives. Impedance spectroscopy provides information with respect to a wide range of frequencies corresponding to different depths of the tissues.

It is performed through a system consisting of an impedance spectroscope, a dedicated anal probe, and a machine-learning module to interpret test results.

This exam represents a novel, non-invasive technique, and it appears to be safe and reproducible, with high sensitivity and specificity in detecting anal sphincter injuries, causing no additional discomfort when compared with physical rectal examination [58,59]. Furthermore, this method can be helpful in the assessment of sphincter impairment sustained by neurogenic damages.

The application of impedance spectroscopy in medical practice must be considered as an early screening method for OASIS, complementing the physical examination, rather than an alternative to EAUS, which remains the gold standard of advanced diagnostics.

A prospective, multicentre study (ONIRY) comparing performances of impedance spectroscopy with those of EAUS and high frequency anal manometry is ongoing.

### 2.2. Undiagnosed OASIS and Anal Incontinence Scores

As stated before, the main risk of an undiscovered sphincter injury is the development of anal incontinence (AI), which can occur long after delivery. AI can range from the involuntary passage of flatus to complete evacuation of liquid or solid faecal matter, thus affecting quality of life with different degrees of severity. The standardized assessment of severity of AI symptoms could be helpful to allow a better evaluation of these patients and to identify those with more severe disease or sphincter defects who may benefit from more invasive procedures. To this purpose, several scoring systems have been proposed. Most of these scores include similar criteria, such as the nature of the incontinence, the degree of loss awareness, the quantity of loss, the frequency of the episodes, and eventual accompanying complaints.

Some of the most widely spread incontinence scores are the Wexner Cleveland Clinic Florida Score [60], the St. Mark’s (Vaizey) Score [61], and the Pescatori Score [62] (Table 2).

## 3. Management

### 3.1. Primary Surgical Repair—Sphincteroplasty

If OASIS is diagnosed following vaginal delivery, surgical repair is carried out as soon as possible after childbirth and is defined as a primary repair, representing the mainstay of treatment. When resources for immediate repair are not available, OASIS repair may be delayed for up to 12 h without apparent detrimental effect [63].

The goal of sphincter repair (either primary or secondary) is to restore a functioning anal canal by reconstruction of a muscular cylinder that is at least 2 cm thick and 3 cm long [64].

Meticulous haemostasis and anatomic approximation with a multilayer closure of all disrupted tissue layers are the key principles for preventing complications and restoring faecal competence.

Tears involving the IAS (grade 3c,4) should be properly identified and repaired separately using the end-to-end technique with interrupted or mattress sutures; reapproximating this layer is important for achieving anal continence.

**Figure 4:**
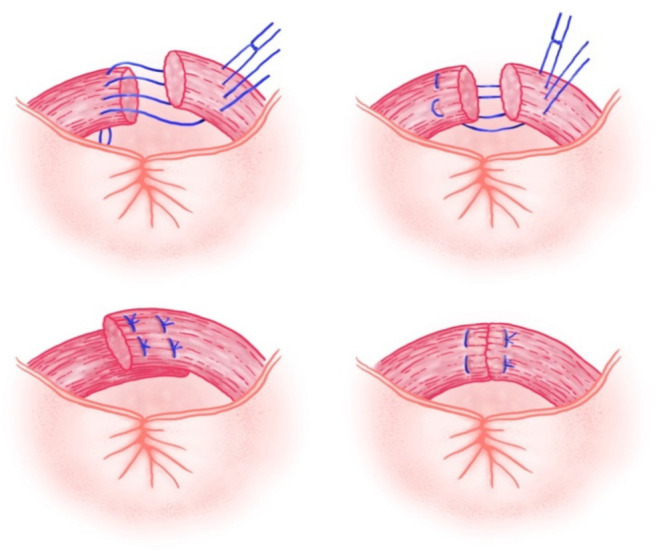
Methods for surgical repair of anal sphincter tears: Overlap technique (images on the left) and end-to-end technique (images on the right).

There are two recognized methods for the repair of a damaged EAS: end-to-end (approximation) and overlap repair (Figure 4).

In a partial thickness EAS tear (grade 3a and partial thickness 3b), an end-to-end repair should be performed by the end-to-end method since the overlapping of partial thickness EAS tears would exert undue tension on the repair. The torn ends of the EAS are approximated and sutured, using interrupted or figure-of-eight sutures (usually at least four or five).

The overlap technique can only be used for full-thickness tears, as two free ends of the muscle are needed for a proper tension-free overlap repair. The torn ends of the EAS are brought together and sutured by overlapping 1 to 1.5 cm of the muscle ends, one over the other, in a double-breasted fashion [65].

A 2013 meta-analysis did not observe significant differences in the overall rate of perineal pain, dyspareunia, flatus incontinence, and FI between the two repair techniques; the overlap group showed significantly lower relative risk of FI at 12 months compared to the end-to-end group [66].

A subsequent randomized trial [67] comparing the two techniques documented worse functional outcomes associated with the overlap technique.

The absence of clear evidence favouring one technique over the other indicates that the practitioner can perform either an end-to-end or overlap repair of the EAS at their clinical discretion.

After anal sphincter repair for OASIS, clinical follow-up at 6 to 12 weeks after delivery is recommended [1]. During this postnatal check, women should be encouraged to report the presence of symptoms of flatus or faecal incontinence, as they may not be prone to reveal such symptoms voluntarily.

When possible, women with incontinence or pain at follow-up should be referred to a regional dedicated multidisciplinary perineal or pelvic floor clinic for endoanal ultrasonography and anorectal manometry; otherwise, referral to a specialist gynaecologists or colorectal surgeon is an appropriate alternative.

### 3.2. Obsteric Trauma-Induced Fecal Incontinence

**Figure 5:**
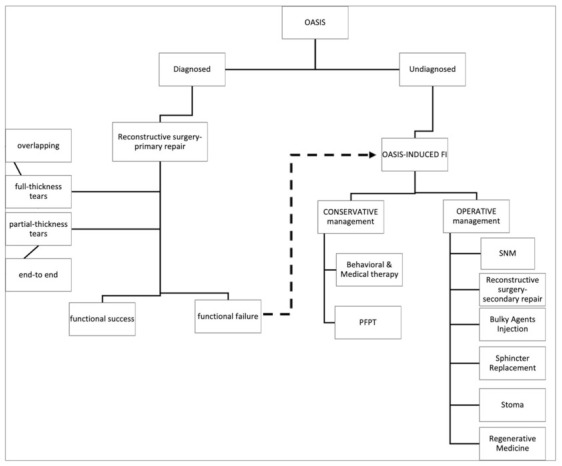
Proposed algorithm for the management of OASIS. OASIS: obstetric anal sphincter injuries. FI: fecal incontinence. PFPT: pelvic floor physical therapy. SNM: sacral nerve modulation.

FI after OASIS can occur several years after the initial sphincter injury. It may result both from an undiagnosed anal sphincter injury and from a functional failure of sphincteroplasty. Regardless of the cause, management of FI is challenging and should be individualized. Treatment options include various conservative and interventional measures (Figure 5). As reported by a recent systematic review, up to 37 different treatments for FI have been studied by RCTs, although none of them persistently improved outcomes, and no treatment ranked best showed superiority for any outcome [68].

#### 3.2.1. Conservative Management

Medical and behavioural measures represent the first-line therapy of FI. Optimization of stool consistency and reduction of bowel motility through changes in dietary habits, fluid intake management, and pharmacological treatments have proven to be effective [4]. Among pharmacological treatments, zinc-aluminium ointment has recently proved to improve the incontinence score and the patient’s FI-quality of life, possibly by increasing the contraction of smooth muscle [68].

Pelvic floor physical therapy (PFPT) is a non-invasive treatment that should be considered as a complementary approach when the aforementioned measures are insufficient.

PFPT may involve manual therapy, biofeedback (a physiotherapy technique that uses external equipment to provide visual or auditory biofeedback during muscle training) or electrical stimulation (ES), behavioural education, and home exercise programs. PFPT has robust, evidence-based support as a first-line, minimally invasive option to treat pelvic floor dysfunction, including pelvic organ prolapse (POP), faecal or urinary incontinence, peripartum and postpartum pelvic floor dysfunction, and chronic pelvic pain [69].

The difficulty in early diagnosis of OASIS often prevents provision of the appropriate treatments in the first 12 h from the lesion occurrence, which represents the timeframe associated with the higher chance of cure. Given the non-invasive nature of PFPT, its low cost, and the possibility to perform it individually at home, it could be reasonable to recommend this treatment to all women at high risk for the development of OASIS.

Although some studies demonstrated that performing individually adapted PFPT on a regular basis may reduce postpartum AI symptoms [70], more evidence is still needed to assess the effectiveness of this therapy in patients with OASIS [71].

Since the internal anal sphincter consists of smooth muscles and is thus not amenable to voluntary exercises, electrical stimulation could represent a strategy to improve the strength and coordination of these smooth and slow muscular components. The underlying mechanism of electrical nerve stimulation possibly involves a neuro-regenerative response through the upregulation of neurotrophic factors in the motoneurons. This technique may therefore improve recovery after childbirth and ameliorate symptoms of faecal or urinary incontinence by promoting neuro-regeneration after injury [72,73].

Of note, the combination of nonsurgical treatments of chronic faecal incontinence, such as electrical stimulation and electromyographic biofeedback (also referred to as triple-target treatment), appeared to be superior to biofeedback alone [73].

#### 3.2.2. Operative Management

Reconstructive surgery, which is carried out several months or years after the initial sphincter injury, is referred to as secondary repair. It follows the same principles of primary repair, and it can be performed either by colorectal surgeons or by appropriately trained gynaecologists. Repeat sphincter repair after a failed primary reconstructive surgery should be considered only if other treatment modalities have been ineffective or if there is an identifiable factor responsible for failure [4].

Sacral nerve modulation (SNM) is a minimally invasive, effective, and sustainable treatment and represents a reasonable first-line surgical option for FI [4].

This technique enhances the impaired sphincter function by continuous, pulsating electrical stimulation of the sacral nerves.

Regardless of FI aetiology (OASIS, pudendal neuropathy, previously failed sphincteroplasty), SNM has been consistently demonstrated to be effective in improving the continence mechanism [68,74].

However, patients should be correctly informed about the common adverse events (in 15% of patients, infection and dislocation being the most frequently observed) and the need of reinterventions in the long term (revision or reimplantation needed in 24% of patients at five years) [75,76].

Perianal injection of bulking agents consists of the inoculation of biocompatible compounds into the submucosal or inter-sphincteric space of the anal canal (i.e., NASHA/Dx injections). It may have some short-term benefit, but to date, little evidence supports its use in the treatment of FI [77].

Artificial bowel sphincter implantation may represent an option of treatment in highly selective patients with severe FI for whom all other treatments have failed.

Currently available implants are fluid-filled cuffs. Artificial bowel sphincters seems as effective as SNS in improving continence and quality of life and superior in improving incontinence scores when compared to placebo, medical management, BF-PFPT plus medical management, tibial nerve stimulation, and other non-surgical treatments [68].

This technique is burdened by a high rate of complications, such as wound infections or post-operative pain and consecutive re-surgeries. Long-term re-operation rates reach 95%, with definitive ex-plantation rates of 40% [78].

Prerequisites for future devices are the ability of adaptation to the bowel pressure changes as well as the long-term performance of the systems.

Faecal diversion with the creation of a colostomy remains an alternative for incontinent patients with a poor quality of life for whom other therapies have failed or are precluded [79].

Other treatments that are not in mainstream use have been described, including radiofrequency energy administration, tibial nerve stimulation, implantation of Tiersch and implantation of magnetic ring, and muscle transposition techniques, such as graciloplasty and gluteoplasty [68,80].

#### 3.2.3. Future Perspectives

Regenerative medicine (RM) strategies are increasingly studied as a future treatment option given the unsatisfactory results of conventional treatments for FI.

The goal of RM as a therapy for FI is to restore the function of the anal sphincter by targeting smooth and skeletal muscle tissues. Among the various approaches in this field, cell therapy, biomaterials, and tissue engineering are the most promising ones.

Cells are transplanted to promote repair and regeneration of tissues both by directly replenishing the tissues and by releasing trophic factors to attract and activate other cells. Biomaterials provide scaffold structure and promote the integration of cells with the host tissue.

Current evidence demonstrates better functional and histological results when cells, scaffolds, growth factors, cytokines, or electrostimulation are combined.

The available literature on this subject includes articles with a wide variation in the studied groups, interventions, and outcome measurements, thus not yet allowing for univocal conclusions [81,82].

## 4. Conclusions

To improve diagnosis and management of OASIS, a widespread awareness among healthcare professionals on this problem and its related consequences is essential.

In the acute setting, thorough examination of the perineum by midwives and physicians is mandatory to assess the anal sphincter for damages.

The use of advanced diagnostic tests as an aid to clinical examination is required to improve prompt diagnosis and to enable immediate and appropriate reconstruction.

Among the latest proposed techniques, impedance spectroscopy is a promising tool for early diagnosis of OASIS, as it can be easily used also by untrained professionals.

During follow-up or in the primary care setting, practitioners should refer women with any symptoms of faecal incontinence and history of obstetric perineal trauma to a dedicated pelvic floor centre or to a gynaecologist or colorectal surgeon.

Since none of the traditional options, both operative and non-operative, for the treatment of obstetric trauma-induced faecal incontinence is fully satisfactory, newer strategies have been proposed. The available preclinical studies suggest a promising beneficial effect of regenerative medicine on FI. Further research in this field is needed to translate regenerative medicine in a concrete alternative for the treatment of FI following postdelivery injuries.

## Figures and Tables

**Table 1:** Comparison between the available diagnostic tools for the assessment of OASIS.

Diagnostic Test	Target	Sensitivity	Accuracy	Reproducibility	Non-Op. Dependence	Intraoperative Use
TRADITIONAL						
EAUS	Morphology	++	++	-	-	+
Anorectal Manometry	Function	+	+	+	+	-
MRI	Morphology	++	++	++	++	-
EXPERIMENTAL						
TPUS	Morphology	+	+	-	-	+
Impedance Spectroscopy	Morphology Function	+	+	++	++	-

**Table 2:** Comparison between the available diagnostic tools for the assessment of OASIS.

Cleveland Clinic Florida (Jorge–Wexner)—Faecal Incontinence Score (CCF-FIS)					
Type of Incontinence	Frequency ^1^				
	Never	Rarely	Sometimes	Usually	Always
Solid	0	1	2	3	4
Liquid	0	1	2	3	4
Gas	0	1	2	3	4
Wears pad	0	1	2	3	4
Lifestyle alteration	0	1	2	3	4
Minimum score 0 (perfect continence), maximum score 20 (complete incontinence)					
**St. Mark’s (Vaizey) Incontinence Score**					
**Type of incontinence**	**Frequency ^1^**				
	Never	Rarely	Sometimes	Usually	Always
Solid	0	1	2	3	4
Liquid	0	1	2	3	4
Gas	0	1	2	3	4
Lifestyle alteration	0	1	2	3	4
				NO	YES
Need to wear a pad or plug				0	2
Taking constipating medicines				0	2
Lack of ability to defer defecation for 15 min				0	4
Minimum score 0 (perfect continence), maximum score 24 (complete incontinence)					
**Pescatori Incontinence Score**					
**Degree**	**Frequency**				
(A) Incontinence for flatus/mucous	Less than once a week				1
	At least once a week				2
	Every day				3
(B) Incontinence for liquid stool	Less than once a week				1
	At least once a week				2
	Every day				3
(C) Incontinence for solid stool	Less than once a week				1
	At least once a week				2
	Every day				3
AI score = AI degree (A = 1, B = 2, or C = 3) + AI frequency; Minimum score is 0, maximum score is 6 (C3).					

^1^ Frequency: Never, 0; Rarely, <1/month; Sometimes, <1/week, ≥1/month; Usually, <1/day, ≥1/week; Always, ≥1/day.
